# Efficiency evaluation on case finding strategy for COVID-19 outbreak control under China’s “dynamic zero-case policy”: a retrospective field epidemiology study

**DOI:** 10.3389/fpubh.2025.1516208

**Published:** 2025-06-13

**Authors:** Zhibei Zheng, Wanwan Sun, Qiuyao Duan, Shelan Liu, Enfu Chen, Jinren Pan

**Affiliations:** ^1^Hangzhou Center for Disease Control and Prevention, Hangzhou, China; ^2^Zhejiang Provincial Center for Disease Control and Prevention, Hangzhou, China; ^3^School of Pubic Heath, Xiamen University, Xiamen, China

**Keywords:** COVID-19, case finding, public health and social measures, emergency response, efficiency of control measures

## Abstract

**Objectives:**

To evaluate the efficiency of case-finding strategy for COVID-19 outbreak control during the “dynamic zero-case policy” period in Zhejiang Province, China, in 2022.

**Methods:**

A field epidemiological observational study was conducted to describe the proportion and time distribution of all cases identified in the event. Categorical data were expressed as counts/proportions or positive rates. The proportion of control lead-time was developed to evaluate the performance of management in various at-risk populations. Positivity rates were used to evaluate the efficiency of certain case-finding approaches. The Pearson χ^2^ test was used to compare proportions between the groups.

**Results:**

Close-contact tracing identified 62.3% of the total cases. Low-risk area resident screening revealed 15.2% of the cases, most of which were ascertained within the first 3 days. All cases found by second-layer contact tracing had a longer control lead-time, but transmission events were believed to occur during the transportation or quarantine period. A higher proportion of positive control lead-time was more effective in curbing SARS-CoV-2 transmission on subsequent days. The proportion of control lead-time between those with and without quarantine status was statistically different (χ^2^_(1)_ = 248.5, *p* < 0.001). Four hundred and forty-two cases (0.571%) were found out of 77,462 close contacts, while 9 cases (0.104%) were found out of 8,683 second-layer contacts (χ^2^_(1)_ = 32.7, *p* < 0.001). On average, 2.2, 7.4, and 106.5 cases were detected per million tests by low-, medium-, and high-risk area residents, respectively.

**Conclusion:**

Rapid and stringent comprehensive public health and social measures can contain the spread of SARS-CoV-2 in a localized area within weeks. Close-contact tracing plays a pivotal role in COVID-19 outbreak control, but contact tracing alone is insufficient to contain the transmission. Mass screening in the early stage and outbreak site exposure person tracing also play an important role for cases finding. It is suggested that in containing severe acute infections with direct transmission route in the future, if stringent social distancing requirements have already being implemented, measures such as tracing second-layer contacts, repeated mass screening for medium-or low-risk residents during the middle and late stages of the event are not recommended.

## 1 Introduction

The coronavirus disease 2019 (COVID-19) pandemic, caused by severe acute respiratory disease coronavirus 2 (SARS-CoV-2), has led to an unprecedented public health crisis. Active case identification, isolation, contact tracing, and quarantine are the backbones of public health and social measures (PHSM) for the early identification of COVID-19 cases to contain SARS-CoV-2 transmission (1). For a long time, before the end of 2022 in China, the so-called “dynamic zero-case policy” was implemented with a rapid and stringent PHSM when indigenous transmission occurred (2–4). The goal of the policy was reducing the number of cases to zero within a localized area as quickly as possible by flexible approaches to identifying and containing new infections.

Case finding is a crucial means for timely detection and control of the source of infection, and it also reflects the field management mode of different populations at-risk. Case finding strategy in infectious disease outbreak refers to a systematic approach or plan designed to identify individuals who are at risk of the disease or already have been infected. However, studies evaluation on efficiency of case finding strategy based on field observational data are still sparse. Numerous studies have used mathematical models to evaluate the impact of intervention measures since the early stages of the COVID-19 pandemic (5–7). Mathematical models can provide useful insights into situational awareness and decision support for policymakers. However, care should be taken to identify the gap between diverse realities and the generalizability of the findings. Most observational studies have only evaluated the effectiveness of contact-tracing programs (8, 9) or compared the outcomes of PHSM between different countries or regions used in ecological studies (10–12). Old-style public health measures can halt an epidemic even when effective medical countermeasures are not available (13); however, many of these interventions involve lockdowns and quarantine measures, which result in high costs to the economy and society (1, 2). Evidence-based public health decision-making is essential to ensure the benefits of intervention measures outweigh their burden (14), and should also emphasize the findings gained from real-world settings.

This study aimed to evaluate the efficiency of case-finding strategy during the China’s “dynamic zero-case policy” period by analyzing the field data from an indigenous COVID-19 outbreak. We describe the proportion and time distribution of the cases identified using different approaches. In particular, we tried to develop “control lead-time” as an indicator for evaluating the performance of management on different at-risk populations. Investigating the efficiency of the case-finding strategy can help make rational risk management recommendations related to exposed individuals, which may support decision-makers in choosing more precise and efficient ways to tackle the next public health emergency.

## 2 Methods

### 2.1 Study design and study setting

We conducted a retrospective observational study to evaluate the efficiency of case-finding approaches during the local COVID-19 outbreak (Omicron variant BA.5.2) in 2022. The outbreak occurred in Yiwu, a county-level city in Zhejiang Province in eastern China, with a population of 2.6 million, and lasted for 21 days (August 2 to August 22, 2022). A total of 710 cases (including 346 asymptomatic infections) were identified in Yiwu, including 342 male and 368 female cases, with a median (percentile 25, percentile 75) age of 35 years (22, 49). Stringent containment measures were quickly implemented when local transmission events were reported, such as case isolation, domestic travel restriction, and tracing and management of exposed individuals. Later on, more rigorous “static social management” measures (including stay-at-home orders, cancelation of any mass gatherings, school closures, etc.) were adopted on August 11, 2022.

A confirmed case was defined as the positive result of the SARS-CoV-2 test by reverse transcription polymerase chain reaction (RT-PCR) in a clinical specimen according to the Chinese “Protocol on the Prevention and Control of COVID-19 (9th Edition)” (15). The case-finding approach was determined based on individual exposure experience and regional risk assessment at the time of diagnosis by thoroughly reviewing case investigation reports. A list of close-contact, second-layer contact, and daily testing numbers for risk area screening was derived from the Comprehensive Integrated Application of Precision and Intelligence for Epidemic Response System developed by the Zhejiang Provincial Government for real-time collection of relevant information on preparedness and response to epidemics.

### 2.2 Outcomes

Case-finding approaches: We divided the case-finding approaches into two categories: quarantine and non-quarantine. The quarantine status categories included close-contact tracing, second-layer contact tracing, outbreak site exposure person tracing, and resident tracing in communities with cluster cases. The not under quarantine status category included high-risk occupational groups (e.g., health care professionals, quarantine facility personnel) screening, high-risk area residents screening, medium-risk area residents screening, low-risk area residents screening, health-seeking patient screening, and others (e.g., self-reported with exposure experiences, with abnormal “health code” in mass screening). Quarantined individuals were mandatory isolation in a quarantine facility or at home for more than 7 days with repeated testing and symptom monitoring. Regions with different transmission risk levels adopted corresponding response measures, such as stay-at-home, lockdown, and multiround screening.

Close contact was defined as close proximity to a COVID-19 patient without proper use of personal protective equipment (PPE) from 2 days before the onset of illness (if the patient was asymptomatic, from 2 days before the patient was sampled) to the date of isolation; explicit duration and distance of exposure were not specified. Second-layer contact was defined as household or social activity exposure with long-term, high-risk close contact (and without contact with the primary patient). High-risk close contact referred to close and prolonged contact with a patient, with exposure usually occurring in the household or workplace. An outbreak site exposure person referred to anyone exposed to a COVID-19 case in a high-risk exposure site (workplace or indoor public place) but did not meet the close-contact definition. Local transmission community residents included those living in communities with clusters of cases. Regarding the risk level of a region, high-risk areas, defined as the neighborhood of confirmed COVID-19 cases/cases of habitual residence, might include several buildings, a residential quarter, or a small village. A medium-risk area was a larger area encircling a high-risk area, usually with boundaries of highroads or rivers. Low-risk areas referred to larger emergency response regions, such as several towns or an entire county. The testing policies were strengthened during the outbreak. Exposed people under quarantine status required daily testing. High-or medium-risk area residents were tested daily in the first 3 days and then adjusted by the field response team based on the risk assessment. The frequency of testing in low-risk areas was also determined by the field response team, which was usually conducted every 3 days. People in the risk area screening might have different repeated testing times according to their compliance, and the daily testing data were collected 7 days after the last case was found. High-risk occupational groups required screening every 2 days.

Control lead-time: Considering the time of the first positive SARS-CoV-2 test as the theoretical quarantine start time, the control lead-time was defined as the time interval from the actual quarantine time to the first positive test time. A control lead-time of <24 h was counted as leading 0 day. For example, if a case was quarantined at 10:00 on August 5 and the first positive test result was reported at 12:00 on August 7, then the control lead-time was calculated as 2 days.

The efficiency of the case-finding approach: The efficiency was evaluated by positive rate of certain case-finding approach. Positive rate = No. Cases ascertained/ No. Exposed individuals or swab tests × 100%.

### 2.3 Statistical analysis

We analyzed information from all the identified cases and their contacts. Categorical data were expressed as counts/proportions or positive rates. The Pearson χ^2^ test was used to compare proportions between the groups. A significance level of less than 0.05 (*p* < 0.05, bilateral) was considered significant, and Bonferroni-adjusted *p* values for multiple comparisons. Analyses were performed using the R statistical software version 4.4.0 (R Foundation for Statistical Computing, Vienna, Austria).

## 3 Results

### 3.1 Case-finding approach

Of 710 cases, 511 (72.0%) were in the quarantine status category. Close-contact tracing was the dominant method of case finding (62.3%, 442 of 710 confirmed cases). Screening of low-risk area residents was the second most important way to identify cases, with 108 cases (15.2%) identified by this method. The third most common approach was to trace the person exposed to the outbreak site, which found 42 cases (5.9%) (Table 1).

**Table 1:** Number of cases found by different case finding approach.

Case finding approach	No. cases (%)	
Under quarantine status	511	(72.0)
Close-contact tracing	442	(62.3)
Outbreak site exposure person tracing	42	(5.9)
Local transmission community residents tracing	18	(2.5)
Second-layer contact tracing	9	(1.3)
Not under quarantine status	199	(28.0)
Low-risk area residents’ screening	108	(15.2)
High-risk occupational group screening	28	(3.9)
Medium-risk area residents screening	26	(3.7)
Health-seeking patient screening	18	(2.5)
High-risk area residents screening	13	(1.8)
Others	6	(0.8)
Total	710	(100.0)

**Figure 1:**
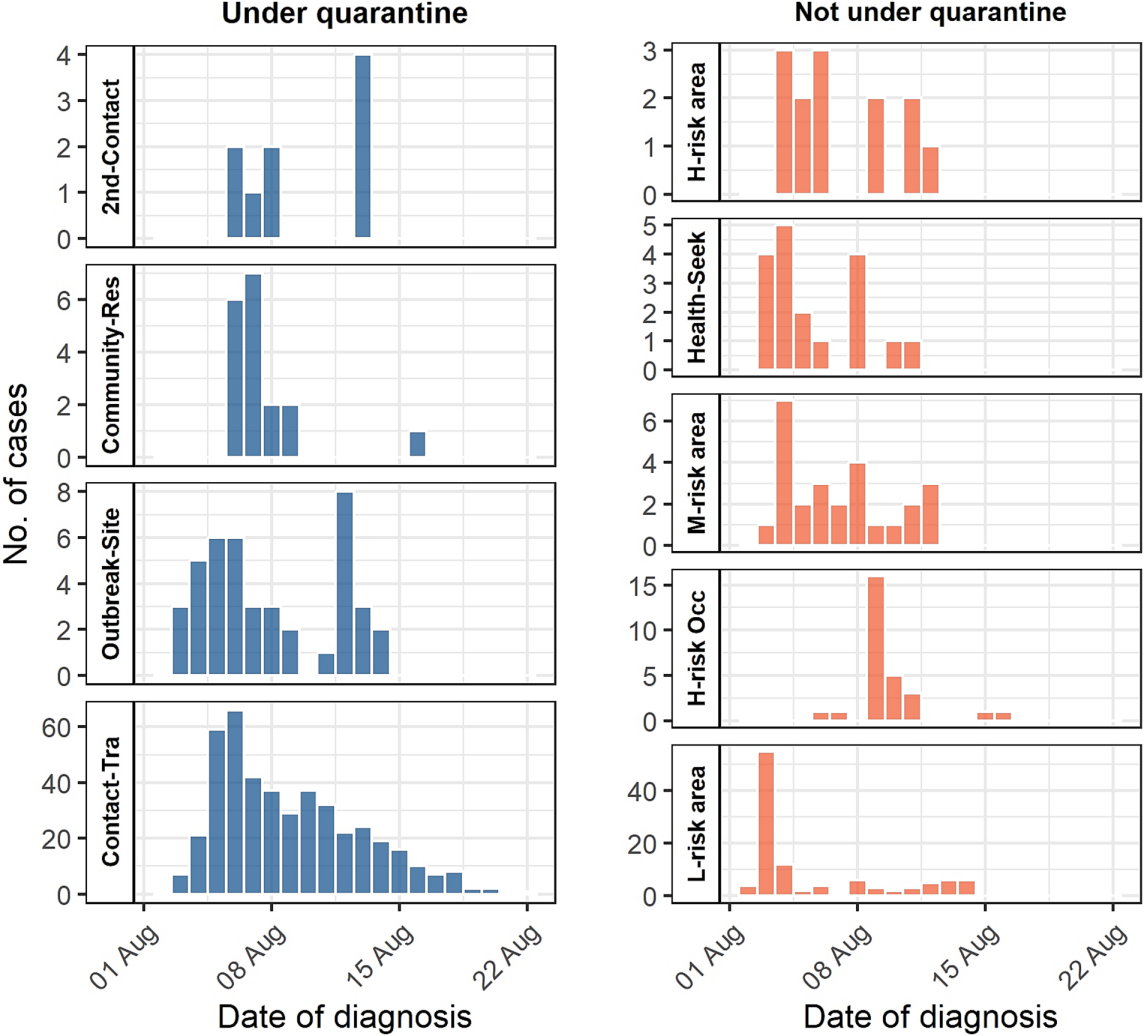
Diagnosis date distribution of case-finding approach. 2nd-Contact = Second-layer contact tracing; Community-Res = Local transmission community residents tracing; Outbreak-Site = Outbreak site exposure person tracing; Contact-Tra = Close-contact tracing; H-risk area = High-risk area residents screening; Health-Seek = Health-seeking patient screening; M-risk area = Medium-risk area residents screening; High-risk Occ = High-risk occupational group screening; L-risk area = Low-risk area residents screening.

Figure 1 shows the time distribution characteristics of the dates of diagnosis using different case-finding approaches. The cases found by close-contact tracing persisted from the 2nd day to the end of the event, with rapid growth in the first 4 days, followed by a longer downtrend. Outbreak site exposure person tracing showed two peaks: early and middle stages of the outbreak. Local transmission community resident tracing occurred mainly from Days 5 to 8. For low-risk area resident screening, 65.7% of the cases were found within the first 3 days, and no further cases were found since day 14. The number of cases identified by screening high-risk area residents, medium-risk area residents, and health-seeking patients was clustered in the early to middle stages.

### 3.2 Control lead-time

**Figure 2:**
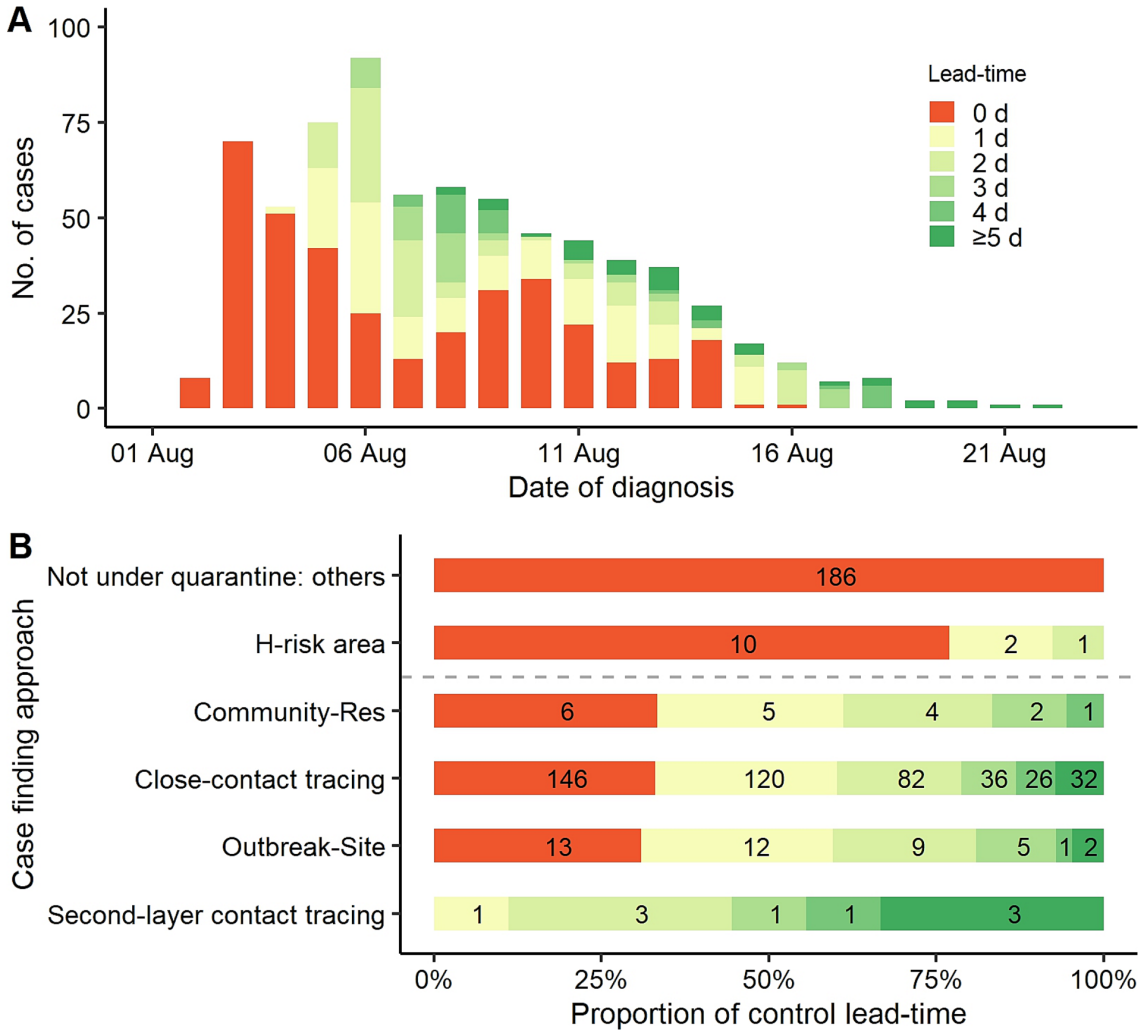
Distribution of control lead-time. **(A)** Control lead-time of daily incidence cases. **(B)** Proportion of control lead-time by different case-finding approaches. Not under quarantine: others = all other approaches not under quarantine status, except for screening high-risk area residents. H-risk area = high-risk area residents screening. Community-Res = Local transmission community residents tracing. Outbreak-Site = Outbreak site exposure person tracing.

Judging from the time series of daily incident cases, a higher proportion of positive control lead-time indicated a decrease in incident cases in the following days and vice versa (Figure 2A). The cases found in the first 2 days (August 2 to August 3, 2022) did not have any lead-time. The proportion of positive lead-time increased significantly in the following 4 days and then fluctuated over time. Since August 17, 2022, all incident cases had two or more days.

Based on the case-finding approaches, the proportions of control lead-times ≥ 1 d were 100.0, 69.0, 67.0, and 66.7% for second-layer contact tracing, outbreak site exposure person tracing, close-contact tracing, and local transmission community residents tracing, respectively. Comparatively, 98.5% of cases found not under quarantine status did not have a positive lead-time, and only 3 of 10 cases had a positive lead-time in high-risk area resident screening (Figure 2B). The proportion of control lead-time between those with and without quarantine status was statistically different (χ^2^_(1)_ = 248.5, *p* < 0.001).

### 3.3 Efficiency of case-finding approaches

Under quarantine status, 442 cases (0.571, 95%*CI*: 0.524–0.633%) were found out of 77,462 close-contacts, while nine cases (0.104, 95%*CI*: 0.055–0.227%) were found out of 8,683 second-layer contacts (χ^2^_(1)_ = 32.7, *p* < 0.001). In not under quarantine status, a total of 49991891swabs were tested for low-risk area residents and found 108 cases, 3,509,949 swabs were tested for medium-risk area residents and found 26 cases, as well as 122,091 swabs were tested for high-risk area residents and found 13 cases (Table 2). Equivalently, 2.2, 7.4, and 106.5 cases were detected per million tests in low-, medium-, and high-risk residents, respectively.

**Table 2:** Number of tracing or screening and cases found of selected population at-risk.

Date	Close-contact		Second-layer contact		Low-risk area residents		Medium-risk area residents		High-risk area residents	
	No. persons ascertained	No. cases ascertained	No. persons ascertained	No. cases ascertained	No. tests screened	No. cases ascertained	No. tests screened	No. cases ascertained	No. tests screened	No. cases ascertained
8/2	396		205		627,445	4			7,734	
8/3	3,848	7	1,044		2,069,740	55		1	826	
8/4	6,821	21	1,609		2,285,988	12	114,373	7	5,773	3
8/5	6,957	59	1,330		2,326,762	2	80,734	2	5,771	2
8/6	6,586	66	1,089	2	2,384,769	4	95,103	3	6,835	3
8/7	5,848	42	348	1	2,461,361		93,560	2	9,764	
8/8	3,167	37	358	2	2,486,023	6	101,637	4	8,748	
8/9	4,276	29	466		2,607,202	3	227,581	1	8,561	2
8/10	3,416	37	227		2,320,932	2	256,222	1	11,168	
8/11	5,062	32	606		2,288,442	3	258,512	2	8,476	2
8/12	5,420	22	148		2,287,881	5	260,686	3	8,739	1
8/13	7,421	24	113	4	2,301,163	6	261,504		9,630	
8/14	6,854	19	188		2,164,181	6	266,311		12,277	
8/15	9,861	16	554		2,257,061		267,867		14,290	
8/16	1,056	10	390		2,215,736		296,615		1,920	
8/17	361	7	5		2,102,401		305,409		541	
8/18	95	8	2		1,680,890		308,841		514	
8/19	17	2	1		1,569,288		310,788		524	
8/20		2			1,924,006		2,951			
8/21		1			1,245,280		867			
8/22		1			1,207,898		148			
8/23					1,086,972		37			
8/24					1,083,155		69			
8/25					1,034,519		13			
8/26					1,028,940					
8/27					951,887					
8/28					997,927		45			
8/29					994,042		76			
Total	77,462	442	8,683	9	49,991,891	108	3,509,949	26	122,091	13

## 4 Discussion

In this study, we retrospectively describe the performance of case-finding approaches in an indigenous COVID-19 outbreak in Zhejiang Province, China, which was the largest outbreak in the province during the “dynamic zero-case policy” period. Close-contact tracing identified 62.3% of the total cases. Low-risk area resident screening revealed 15.2% of the cases, 65.7% of which were ascertained within the first 3 days. The third most common approach was outbreak site exposure person tracing, which found 5.9% of the cases. A higher proportion of positive control lead-time was inversely correlated with onward transmission, and there were significant differences between the case-finding approaches. Screening for low-or medium-risk area residents revealed inefficient of case identification, particularly during the middle and late stages of the event.

COVID-19 vaccines confer limited protection against infections caused by the Omicron variant, highlighting the pivotal role of non-pharmacological public health measures in fight against the COVID-19 outbreak (13). An assessment study of the first few waves of the epidemic in four Asian countries showed that case identification and management, coupled with close-contact tracing and isolation, was a successful strategy to contain transmission, whereas social distancing was an effective measure only if strictly and persistently enforced (16). Analysis of the distribution of cases found by different approaches, our study shows clearly that close-contact tracing is one of the most important measures for outbreak control, but for containing the COVID-19 outbreak, other emergency measures are also needed for all exposed individuals. During this outbreak, 37.7% of the cases were detected by means other than close contact tracing. For settings in which transmission events have been proven to occur, it is proposed to trace the outbreak focus sites exposure persons, even without clear clues of contact with an index case. A quantitative study on the effects of public health measures for zeroing the COVID-19 outbreak in China showed that contact tracing was crucial for containing outbreaks during the initial phases, whereas social distancing measures became increasingly prominent as the spread persisted (17). In addition, the active monitoring of potentially exposed individuals (non-close contacts) plays a critical role in identifying COVID-19 cases. In the absence of local transmission in Shanghai from January to February 2020, cases identified by screening individuals from high-risk areas were equivalent to those identified by contact tracing (18).

Second-layer contact tracing was adopted as a control package in China in September 2020. Disappointingly, consistent with our previous findings (19), both field observational evidences showed that second-layer contact quarantine did not have a positive impact on outbreak control. Although all cases found by second-layer contact tracing had a longer control lead-time, the transmission events were believed to occur during the transportation or quarantine period. Through a thorough review of the case investigation reports, we found that of the nine cases found by second-layer contact tracing, eight of their related primary contacts were not infected during the outbreak. Only one pair of cases (both the related primary contact and this second-layer contact were infected) was from the same family, and the date of diagnosis was only one day apart. Namely, only one of the nine second-layer contact cases in which the possible infector was the primary close contact. A retrospective cohort study of field data from a county health department in Germany in 2020 found 21 tertiary cases among 179 (11.7%) second-layer contacts placed in quarantine. The efficacy of quarantine in second-layer contacts was 51.5% of that in primary contacts; however, this study did not provide information on the source of infection in second-layer contact cases (20). An individual-based modeling simulation study showed that the isolation of cases and quarantine of close contacts would not eliminate the local transmission of COVID-19, and quarantine of second-layer contacts could significantly reduce the final infected population size and peak of daily incident cases (21). Another social network simulation model showed that second-layer contact tracing reduced the size of outbreaks more than contact tracing alone. Adding second-layer contact tracing resulted in a smaller percentage (16%) of the population being infected after 70 days, whereas only primary contact tracing resulted in a 48% infection rate. However, second-layer contact tracing led to almost half of the local population being quarantined at a single point in time, similar to the introduction of local lockdown (22). Therefore, our view on second-layer contact tracing is that it can serve as a reinforced intervention in the early stage of an outbreak with only a few cases. It should not be regarded as a routine measure in a large-scale outbreak or as a stay-at-home order implemented in emergency response.

Mass screening also plays a vital role in identifying high-risk areas and populations, particularly in the early stages of an outbreak. During this outbreak, 108 cases were identified by low-risk area resident screening, most of which were ascertained in the first 3 days. Multiround mass screening to identify potential cases is one of the most important public health emergency responses in China (23). However, this study showed low efficiency of mass screening for community residents without considering exposure experience, especially in the middle and late stages of the outbreak. To identify a case of this outbreak, 9,392, 134,998, and 462,888 screening samples were tested in high-, medium-, and low-risk areas, respectively. Since transmission can also be achieved by asymptomatic and presymptomatic infections, implementation of PHSM is necessary to limit social contact (24). Nevertheless, mild restrictions could only slow down onward transmission rather than contain the epidemic (25). In the emergency response process, mass screening led to a large crowd of people gathering for repeated sampling, which seriously weakened the effectiveness of the social distancing measures. Therefore, for the effective containment of an outbreak, it might be necessary to perform mass screening in certain areas at the beginning of the event. However, it should be well organized and adjust timely.

Finally, the proportion of control lead-time developed in this study can serve as a process indicator of the performance of the control measures. The cases found under quarantine status had a larger proportion of positive control lead-times. A larger proportion of the positive control lead-time was more effective in curbing SARS-CoV-2 transmission in the subsequent days.

Both the COVID-19 disease itself and pandemic response activities have led unprecedented impacts on communities, health care providers or families (26–28). Although the impact of the pandemic is gradually fading away, studies evaluating the efficiency and economics of containment measures based on field data are still insufficient. It is suggested to carry out additional researches focusing on those subjects as preparedness for next potential threat.

Our study had some limitations. We primarily used descriptive methods, with a lack of association effect size. As this was a retrospective study, we failed to collect data on the size of some at-risk populations, such as those outbreak site exposure person and high-risk occupational groups. The numbers of residents in the medium-or high-risk were varying over time; therefore, the efficiency evaluation was based on the total number of screening tests.

In conclusion, close-contact tracing plays a pivotal role in COVID-19 outbreak control. However, contact tracing alone is insufficient to contain the transmission. It is not recommended to trace second-layer contacts after stringent social distancing requirements implemented in outbreak response. Mass screening plays a certain role in the early stages of an outbreak for recognizing high-risk areas or populations, but attention should be paid to the issue of a large gap in efficiency between risk areas. Repeated mass screening appears unnecessary for medium-or low-risk residents during the middle and late stages of an outbreak. Outbreak site exposure person tracing also plays an important role for cases finding. Rapid and stringent comprehensive public health and social measures can contain the spread of SARS-CoV-2 in a localized area within weeks. These experiences and lessons can also be referenced in containing other infectious diseases transmitted by either direct contact or direct spread of droplets.

## Data Availability

The raw data supporting the conclusions of this article will be made available by the authors, without undue reservation.
